# Cardiolipin remodeling by ALCAT1 links mitochondrial dysfunction to Parkinson’s diseases

**DOI:** 10.1111/acel.12941

**Published:** 2019-03-05

**Authors:** Chengjie Song, Jun Zhang, Shasha Qi, Zhen Liu, Xiaoyang Zhang, Yue Zheng, John‐Paul Andersen, Weiping Zhang, Randy Strong, Paul Anthony Martinez, Nicolas Musi, Jia Nie, Yuguang Shi

**Affiliations:** ^1^ Department of Biochemistry and Molecular Biology, School of Basic Medical Sciences Nanjing Medical University Nanjing China; ^2^ Barshop Institute for Longevity and Aging Studies, Department of Pharmacology University of Texas Health Science Center San Antonio Texas; ^3^ Perenna Pharmceuticals Inc San Antonio Texas; ^4^ Department of Pathophysiology the Second Military Medical University Shanghai China

**Keywords:** α‐synuclein, cardiolipin, mitochondrial dysfunction, mitophagy, MPTP

## Abstract

Cardiolipin (CL) is a mitochondrial signature phospholipid that is required for membrane structure, respiration, dynamics, and mitophagy. Oxidative damage of CL by reactive oxygen species is implicated in the pathogenesis of Parkinson's disease (PD), but the underlying cause remains elusive. This work investigated the role of ALCAT1, an acyltransferase that catalyzes pathological remodeling of CL in various aging‐related diseases, in a mouse model of PD induced by 1‐methyl‐4‐phenyl‐1,2,4,6‐tetrahydropyridine (MPTP). We show that MPTP treatment caused oxidative stress, mtDNA mutations, and mitochondrial dysfunction in the midbrain. In contrast, ablation of the *ALCAT1* gene or pharmacological inhibition of ALCAT1 prevented MPTP‐induced neurotoxicity, apoptosis, and motor deficits. ALCAT1 deficiency also mitigated mitochondrial dysfunction by modulating DRP1 translocation to the mitochondria. Moreover, pharmacological inhibition of ALCAT1 significantly improved mitophagy by promoting the recruitment of Parkin to dysfunctional mitochondria. Finally, ALCAT1 expression was upregulated by MPTP and by α‐synucleinopathy, a key hallmark of PD, whereas ALCAT1 deficiency prevented α‐synuclein oligomerization and S‐129 phosphorylation, implicating a key role of ALCAT1 in the etiology of mouse models of PD. Together, these findings identify ALCAT1 as a novel drug target for the treatment of PD.

## 1 INTRODUCTION

It is estimated that more than 10 million people are currently affected by Parkinson's disease (PD) worldwide. PD is an aging‐related neurodegenerative disease on the rise due to an increase in the aging population in the United States and developed nations. There is no effective treatment for PD, because the molecular mechanisms underlying the pathogenesis remain poorly understood. Although the precise pathogenic mechanism leading to neurodegeneration in PD is unknown, oxidative stress, mitochondrial dysfunction, and defective mitophagy are all considered as the primary pathogenic mechanisms of cell death of nigral DA and substantia nigra pars compacta (SNpc) neurons, as demonstrated by the neurotoxic effects of 1‐methyl‐4‐phenylpyridinium (MPP^+^), rotenone, paraquat, and other pesticides (Kamel, 2013), all of which cause significant production of reactive oxygen species (ROS), mitochondrial dysfunction, and apoptosis (Chen & Dorn, 2013).

Cardiolipin (CL) is a mitochondrial signature phospholipid that plays a pivotal role in maintaining mitochondrial function, including membrane structure, oxidative phosphorylation, ATP production, mtDNA biogenesis, mitochondrial fusion, fission, and autophagy (mitophagy). CL is highly sensitive to oxidative damage of its four fatty acyl chains by ROS, due to its high content of polyunsaturated fatty acids and exclusive location in mitochondria which are the primary intracellular source of ROS production (Lin & Beal, 2006). Aging is associated with oxidative stress and CL depletion (Hsu & Shi, 2017), which are also implicated in the pathogenesis of PD (Ghio, Kamp, Cauchi, Giese, & Vassallo, 2016). CL oxidation impairs mitochondrial complex I activity, which is implicated in neurotoxicity and apoptosis in 1‐methyl‐4‐phenyl‐1,2,4,6‐tetrahydropyridine (MPTP) mice (Perier et al., 2005). In direct support of a causative role of CL peroxidation in the development of PD, mitochondrial‐targeted antioxidants attenuate pathogenesis and protect CL from oxidative damage in mouse models of neurodegenerative diseases (Fouret et al., 2015; McManus, Murphy, & Franklin, 2011; Skulachev et al., 2010).

Cardiolipin is also required for the various stages of the autophagic process, from autophagosome biogenesis to lysosomal function (Hsu et al., 2015; Hsu & Shi, 2017). Autophagy is required for the mitochondrial quality control process by engaging in crosstalk with ROS to eliminate damaged mitochondria through mitophagy (Hsu & Shi, 2017; Wang et al., 2015). A recent study also showed that constitutive activation of autophagy significantly increases lifespan of mice (Fernandez et al., 2018). Emerging evidence suggests that dysregulation of autophagy may also contribute to the pathogenic process in PD, as evidenced by mouse models of PD and human PD patients with mutations of the *Parkin* and *PINK1* genes, two key regulators of mitophagy (Pickrell & Youle, 2015). PINK1 mutations also cause oxidative stress and render neuronal cells highly sensitive to stress‐induced mitochondrial dysfunction and apoptosis (Chu, Bayir, & Kagan, 2014; Pickrell & Youle, 2015). Additionally, PINK1 mutations lead to lower levels of CL in mitochondria, whereas restoration of CL prevents mitochondrial dysfunction in flies by promoting electron transport between ubiquinone and complex I (Vos et al., 2017). Moreover, CL deficiency has also been implicated in aging and other aging‐related neurological diseases (Hsu & Shi, 2017; Paradies, Petrosillo, Paradies, & Ruggiero, 2011; Shi, 2010). However, the underlying causes for CL peroxidation in PD remain elusive.

ALCAT1 is an acyltransferase that catalyzes resynthesis of CL from lysocardiolipin, a key step involved in the remodeling of CL (Cao, Liu, Lockwood, Burn, & Shi, 2004). Our recent work showed that CL remodeling by ALCAT1 plays a key role in promoting oxidative stress by catalyzing the remodeling of CL with docosahexaenoic acid (DHA) and arachidonic acid (AA) (Li et al., 2010). DHA and AA are enriched with double bonds which render CL highly sensitive to oxidation by ROS. CL oxidation generates lipid peroxides, a more stable form of ROS, leading sequentially to exacerbation of oxidative stress, CL peroxidation and depletion, and mitochondrial dysfunction. Our recent work further demonstrated that upregulated ALCAT1 expression by ROS plays a pivotal role in mitochondrial dysfunction associated with various aging‐related metabolic diseases. Consequently, targeted inactivation of ALCAT1 prevents the onset of various aging‐related diseases, including obesity, type 2 diabetes, and cardiovascular diseases (Liet al., 2012, 2010; Liu et al., 2012; Wang et al., 2015). CL remodeling by ALCAT1 also leads to multiple metabolic defects that are highly reminiscent of those noted in PD, including oxidative stress, mtDNA mutations, and mitochondrial dysfunction. However, whether ALCAT1 is also involved in other aging‐related diseases remains unknown. Using mice with targeted deletion of ALCAT1, we investigated a role for the enzyme in regulating the onset of MPTP‐induced PD. We show that upregulated ALCAT1 expression by MPTP and synucleinopathy, a hallmark of PD, leads to severe oxidative stress, mtDNA mutations, and mitochondrial dysfunction in the brain. Ablation of ALCAT1 or pharmacological inhibition of ALCAT1 prevented the onset of MPTP‐induced neurotoxicity and locomotive defects, implicating a key role of the enzyme in the pathogenesis of PD.

## 2 RESULTS

### 2.1 Ablation of ALCAT1 prevents MPTP‐induced impairment in locomotor behaviors

**Figure 1:**
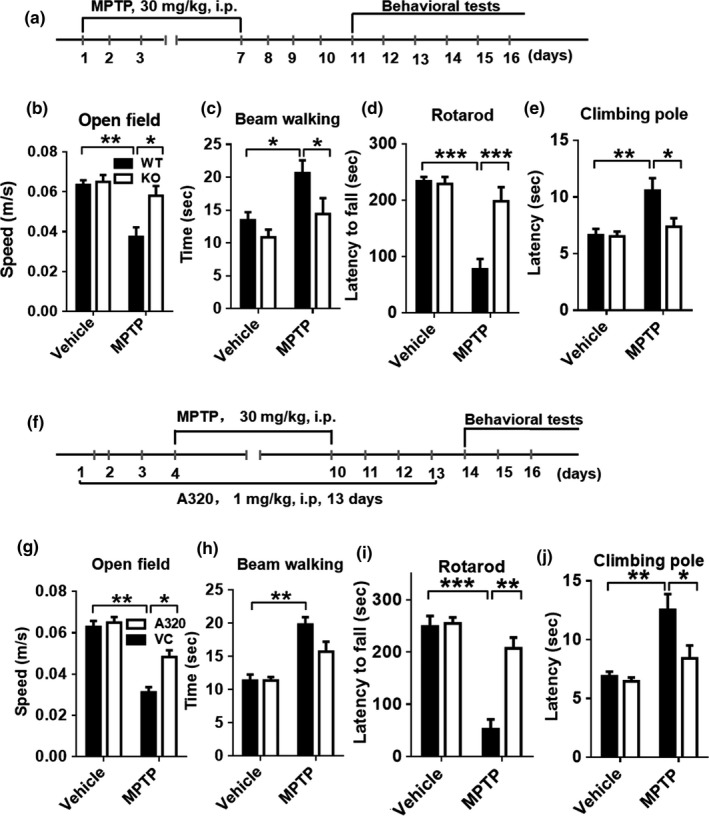
ALCAT1 deficiency or inhibition by A320 protects mice from 1‐methyl‐4‐phenyl‐1,2,4,6‐tetrahydropyridine (MPTP)‐induced motor deficits. (a) Male *ALCAT1^−/−^* mice and wild‐type (*WT*) controls were injected intraperitoneally (i.p.) with MPTP at 30 mg/kg or saline for 7 days, followed by behavioral tests for four consecutive days after the final injection, including: (b) travel speed (m/s) during 6 min; (c) time (sec) to traverse the beam; (d) latency (sec) to fall off the rotated rod; and (e) time (sec) to climb pole. (f) Male C57BL/6 mice were injected i.p. with A320 at 1 mg/kg or vehicle for 3 days prior to the first dose of MPTP and continued for 13 consecutive days. Behavioral tests were performed on days 4–11 after the last dose of MPTP injection, including: (g) travel speed (m/s) during 6 min; (h) time (sec) to traverse the beam; (i) latency (sec) to fall off the rotated rod; (j) time (sec) to climb pole. Data are mean ± *SEM*; *n* = 10, *p < 0.05, **p < 0.01, and ***p < 0.001

Upregulated ALCAT1 mRNA and protein expression have implicated in the pathogenesis of several aging‐related metabolic diseases by catalyzing pathological remodeling of CL with a high peroxidation index (Li et al., 2012). Using mice with targeted deletion of the *ALCAT1* gene (Li et al., 2010), we investigated the role of ALCAT1 in the development of PD in mice treated with MPTP. Male ALCAT1 knockout mice (*ALCAT1^−/−^*) and the wild‐type controls (*WT*) (*n* = 10, 10 weeks) were treated with MPTP by i.p. injection at 30 mg/kg for seven consecutive days and then analyzed for changes in locomotor activity on days 4–11 after the final dose of MPTP injection (Figure 1a). MPTP treatment caused a significant impairment in locomotor activities and coordination skills in *WT* control mice, as evidenced by results from behavior tests, including travel speed (Figure 1b), beam walking (Figure 1c), rotarod (Figure 1d), and pole climbing (Figure 1e). In contrast, these defects were significantly attenuated by ALCAT1 deficiency. The *WT* mice were indistinguishable from the *ALCAT1^−/−^* mice in the vehicle‐treated group, suggesting that ALCAT1 deficiency alone did not change locomotor behaviors.

### 2.2 Pharmacological inhibition of ALCAT1 alleviates MPTP‐induced motor deficits

The *ALCAT1^−/−^* mice are deficient in ALCAT1 expression from embryonic day one, raising an intriguing question whether the protective effect against MPTP‐induced motor deficits could be caused by compensatory responses during development. To address this issue, we next tested the effect of A320, a potent ALCAT1 inhibitor with IC_50_ = 0.1 µM in a cell‐based assay, on MPTP‐induced motor deficits. C57BL/6 mice (10 weeks) were treated with A320 (1 mg/kg, i.p.) 3 days prior to the first dose of MPTP for 13 consecutive days (Figure 1f), followed by analysis of locomotor activities on day 14. Consistent with the findings from the *ALCAT1^−/−^* mice, treatment with A320 significantly attenuated motor deficits of the MPTP‐treated mice, as evidenced by a significant improvement in locomotor activities (Figure 1g) and coordination skills (Figure 1h–j).

### 2.3 ALCAT1 deficiency and pharmacological inhibition attenuate MPTP‐induced DA neuronal degeneration

**Figure 2:**
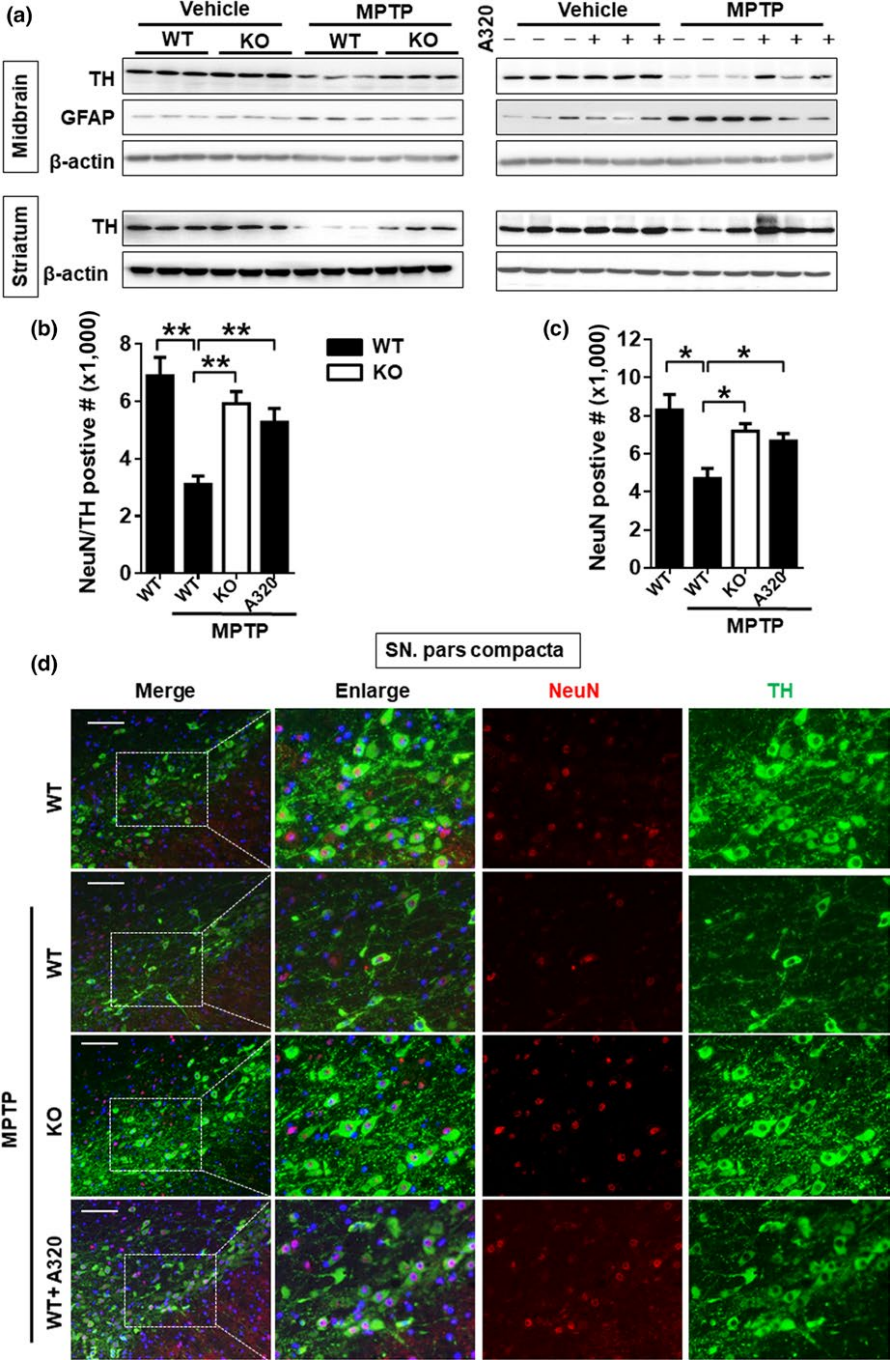
ALCAT1 deficiency or inhibition by A320 attenuated the 1‐methyl‐4‐phenyl‐1,2,4,6‐tetrahydropyridine (MPTP)‐induced neurodegeneration and gliosis. Mice were treated as described in Figure 1, followed by the following analysis: (a) western blot analysis of tyrosine hydroxylase (TH) and glial fibrillary acidic protein (GFAP) protein expression in the midbrain and striatum, *n* = 3; (b) quantitative analysis of the number of TH‐positive neurons in the midbrain; and (c) quantitative analysis of the number of NeuN‐positive neurons in the midbrain. Data are mean ± *SEM*; *n* = 5, *p < 0.05, **p < 0.01. (d) Immunofluorescent imaging analysis of DA neurons in the midbrain from *ALCAT1^−/−^* (KO) and *WT *controls. Tissue samples were immunoblotted with anti‐TH (green) and NeuN (red) antis and DAPI (blue) for nucleus staining, followed by confocal imaging analysis of DA‐positive neurons (TH) and total neurons (NeuN) in the substantia nigra pars compacta (SNpc) area of each mouse. A representative photomicrograph is shown (Scale bars: 100 μm)

A reduction in tyrosine hydroxylase (TH) level in DA neurons is a major defect associated with PD and the main cause of behavioral disorders. Accordingly, we show that MPTP treatment depleted TH protein expression and increased expression of glial fibrillary acidic protein (GFAP), an astrocyte marker that is upregulated in human PD patients (Clairembault et al., 2014), in the midbrain and striatum of *WT* control mice, as evidenced by results from western blot analysis (Figure 2a, left panel, quantified in Supporting Information Figure S1a,c,e). Accordingly, MPTP treatment selectively caused depletion of DA neurons, as evidenced by results from co‐immunohistochemical staining of TH with the pan neuronal marker (NeuN) in SNpc (Figure 2d, quantified in Figure 2b). The depletion of DA neurons also led to a significant decrease in the total number of neurons in SNpc (Figure 2d, quantified in Figure 2c). Consistent with the improved locomotor activity in MPTP mice, ALCAT1 deficiency and inhibition by A320 not only restored TH expression level (Figure 2a, right panel, quantified in Supporting Information Figure S1b,d,f), but also prevented the loss of DA neurons in SNpc (Figure 2d, quantified in Figure 2b).

### 2.4 Upregulation of ALCAT1 by α‐synucleinopathy links MPTP neurotoxicity to apoptosis of neuronal cells

**Figure 3:**
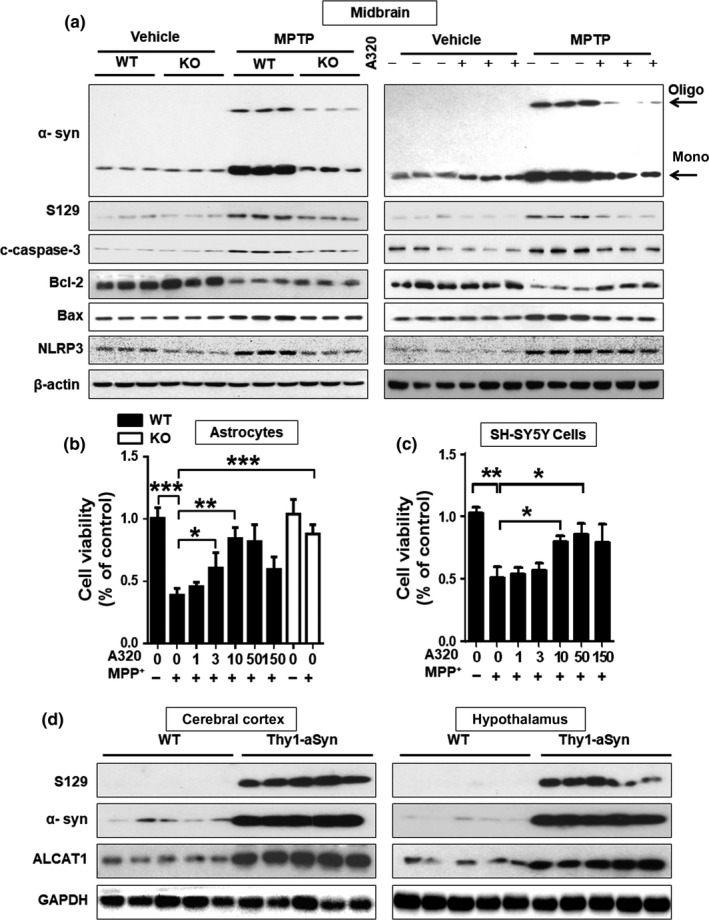
ALCAT1 deficiency or inhibition by A320 attenuates 1‐methyl‐4‐phenyl‐1,2,4,6‐tetrahydropyridine (MPTP)‐induced α‐synucleinopathy and apoptosis. (a) Western blot analysis of protein expression levels of α‐synuclein, S‐129 phosphorylation (phosphor S129), cleaved caspase‐3, Bcl‐2, Bax, and NLRP3 using tissue samples from the midbrain of *WT* and *ALCAT1^−/−^* mice treated with MPTP or vehicle, *n* = 3. (b, c) Primary astrocytes cells (b) and SH‐SY5Y cells (c) were treated with 0.2 mM or 0.5 mM MPP^+^, respectively, in the presence or absence of the indicated concentrations of A320 for 24 hr, followed by analysis of cell viability using a CCK8 assay kit. Data are mean ± *SEM*; one‐way ANOVA, *n* = 9, *p < 0.05, **p < 0.01, and ***p < 0.001. (d) Western blot analysis of ALCAT1 protein expression level in the cerebral cortex and hypothalamus of Thy1‐α‐synuclein transgenic mice and the WT controls, *n* = 5

Oligomerization and S129 phosphorylation of α‐synuclein, the primary component of Lewy body, are a critical hallmark of PD. CL on the outer mitochondrial membrane plays a key role in regulating α‐synuclein stability (Ryan et al., 2018). Consistent with this notion, MPTP treatment stimulated α‐synuclein expression, oligomerization, and S129 phosphorylation in the midbrain of MPTP mice (Figure 3a, quantified in Supporting Information Figure S2a–f). In contrast, ALCAT1 deficiency or inhibition by A320 not only downregulated α‐synuclein expression, but also significantly prevented oligomerization and S129 phosphorylation. Oligomerization of α‐synuclein in response to oxidative stress is also implicated in neuronal death (Souza, Giasson, Chen, Lee, & Ischiropoulos, 2000). Accordingly, MPTP treatment significantly upregulated apoptotic stimulators, including cleaved caspase‐3, Bax, and inflammatory regulator NLRP3, and downregulated the expression of Bcl2, an antiapoptotic regulator in the midbrain. These defects were mitigated by ALCAT1 deficiency and inhibition by A320 (Figure 3a and quantified in Supporting Information Figure S2g–n). Accordingly, treatment of primary astrocytes and SH‐SY5Y neuronal cells with A320 prevented cellular death in response to treatment of 1‐methyl‐4‐phenylpyridinium (MPP^+^), a toxic metabolite of MPTP (Figure 3b,c). We next determine a role of ALCAT1 in mediating a‐synuclein‐induced neurotoxicity in Thy1‐αSyn transgenic mice, a mouse model of progressive PD (Chesselet et al., 2012). Remarkably, ALCAT1 protein expression was significantly upregulated by overexpression of the human α‐synuclein protein in the Thy1‐αSyn transgenic mice, implicating a role for ALCAT1 in the etiology of mouse models of PD (Figure 3d, quantified in Supporting Information Figure S2o,p).

### 2.5 ALCAT1 inhibitor improves mitophagy by promoting Parkin association with mitochondria

**Figure 4:**
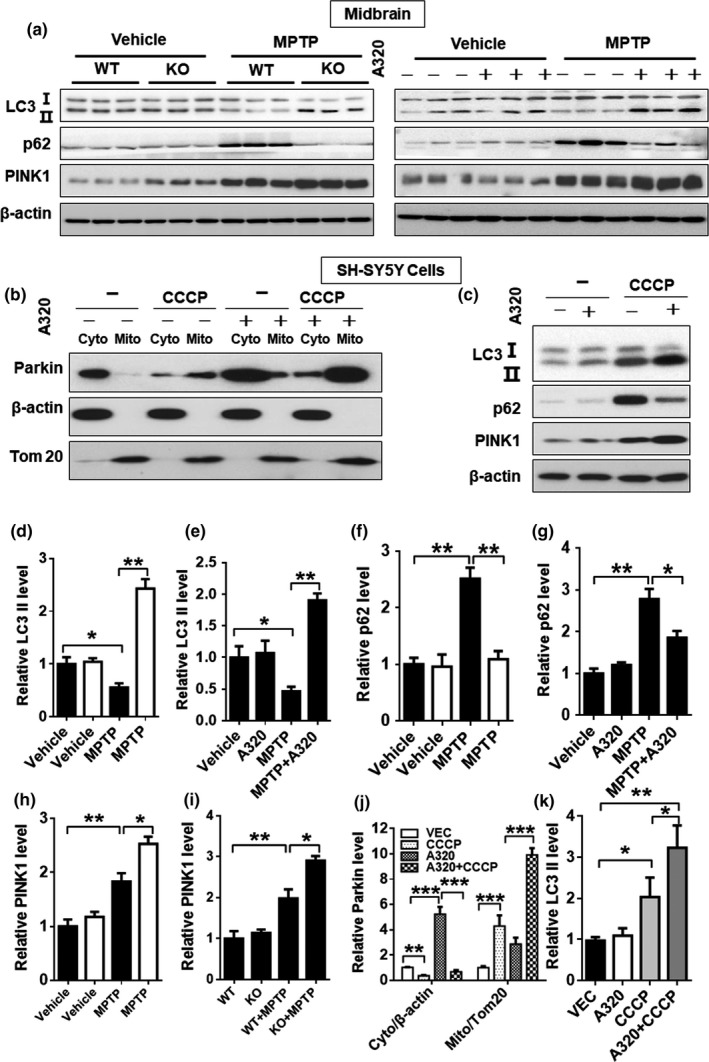
ALCAT1 deficiency or inhibition by A320 mitigates autophagic defects caused by 1‐methyl‐4‐phenyl‐1,2,4,6‐tetrahydropyridine (MPTP). (a) Western blot analysis of protein expression levels of major autophagic regulators, including LC3, p62, and PINK1, in the midbrain of *ALCAT1^−/− ^*(KO) and WT controls in response to treatment with MPTP or vehicle. (b) SH‐SY5Y cells were treated with 10 µM of CCCP in the presence or absence of 10 µM of A320 for 24 hr, followed by subcellular fractionation and western blot analysis of Parkin in each fraction using Tom20 and β‐actin as internal control for mitochondrial and cytosol fractions, respectively. (c) SH‐SY5Y cells were treated with 10 µM of CCCP in the presence or absence of 10 µM of A320 for 24 hr, followed by western blot analysis of protein expression levels of LC3, p62, and PINK1 using β‐actin as an internal control for protein loading. (d–i) Quantitative analysis of relative protein levels in panel a. (j) Quantitative analysis of relative protein levels in panel b. (k) Quantitative analysis of LC3 protein levels in panel c. Data are mean ± *SEM*, one‐way ANOVA, *n* = 3, *p < 0.05, **p < 0.01, and ***p < 0.001

Dysregulation of the autophagic pathway is implicated in the pathogenesis of PD (Lynch‐Day, Mao, Wang, Zhao, & Klionsky, 2012). Consistent with this notion, we found that MPTP treatment significantly increased expression of p62 and decreased expression of LC3II, the lipidated form required for autophagic initiation, suggesting a defect in autophagy (Figure 4a, quantified in Figure 4d–g). Consistent with the notion, ALCAT1 depletion or inhibition by A320 not only normalized the expression of p62 and LC3II, but also significantly upregulated PINK1 expression in the midbrain of MPTP mice (Figure 4a, quantified in Figure 4h,i). PINK1 plays a key role in initiating mitophagy by activating Parkin's E3 ubiquitin ligase activity, leading to the recruitment of Parkin to the dysfunctional mitochondria (Pickrell & Youle, 2015). Accordingly, we further showed that inhibition of ALCAT1 by A320 not only stimulated Parkin expression, but also promoted Parkin association with mitochondria in response to treatment with CCCP, a mitochondrial uncoupler, in SH‐SY5Y neuronal cells (Figure 4b, quantified in Figure 4j). Consistent with the findings, ALCAT1 inhibition by A320 significantly improved mitophagy, as evidenced by decreased p62 expression as well as increased LC3II and PINK1 expression in SH‐SY5Y neuronal cells in response to treatment with CCCP (Figure 4c, quantified in Figure 4k).

### 2.6 Upregulation of ALCAT1 expression by MPTP causes mitochondrial fragmentation by promoting DRP1 association with mitochondria

**Figure 5:**
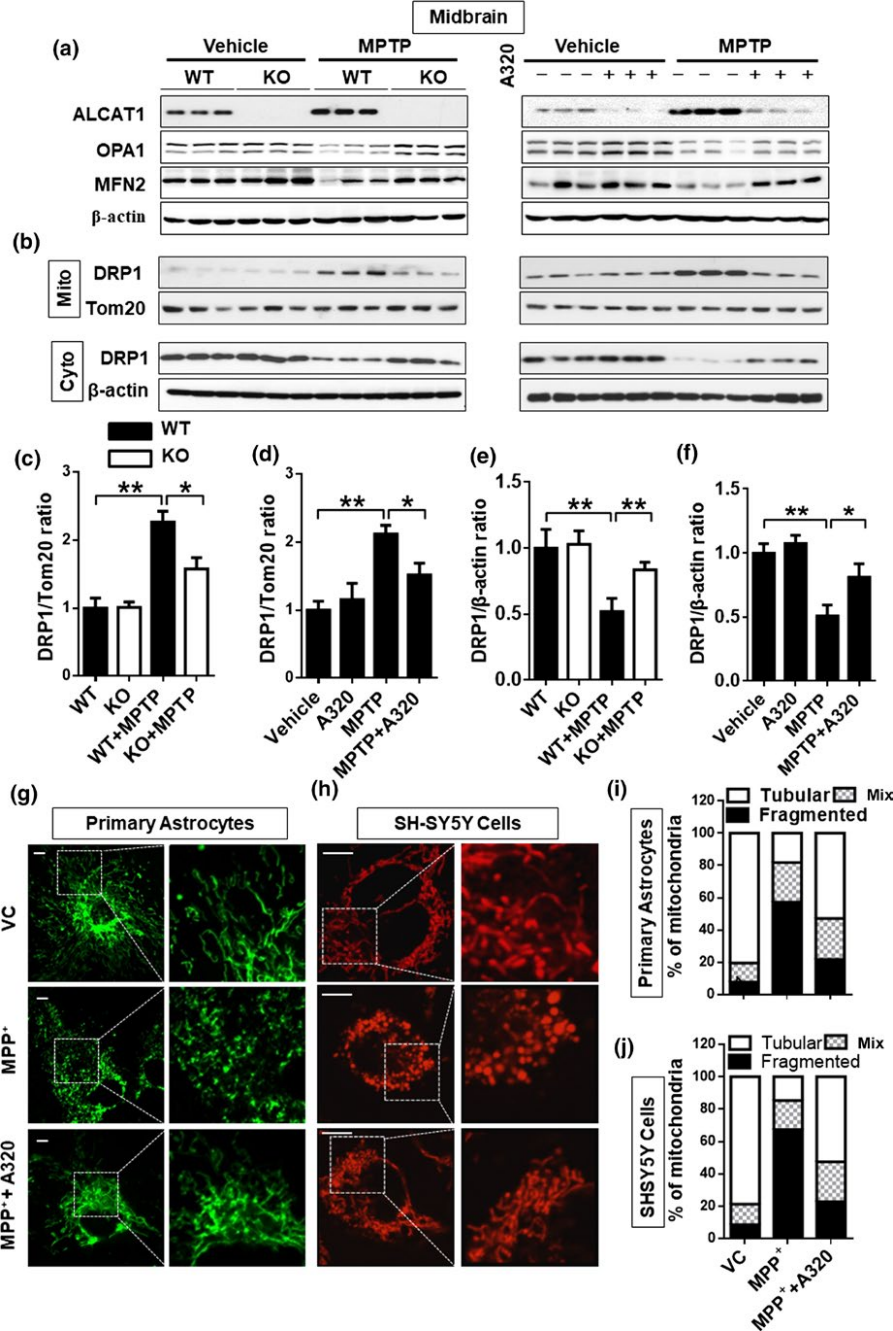
ALCAT1 deficiency or inhibition by A320 prevents 1‐methyl‐4‐phenyl‐1,2,4,6‐tetrahydropyridine (MPTP)‐induced mitochondrial fragmentation in the midbrain by preventing DRP1 translocation to mitochondria. (a) Western blot analysis of protein expression levels of major regulator of mitochondrial fusion and fission, including OPA1 and MFN2, in the midbrain of *ALCAT1^−/−^* (KO) and *WT* control mice (left panel), or in WT mice treated with A320 (right panel). (b) Tissue samples from midbrain were fractionated into mitochondrial and cytosol fractions, followed by western blot analysis of DRP1 protein level in each fraction using Tom20 and β‐actin as internal control for mitochondrial and cytosol fractions, respectively. (c–f) Quantitative analysis of relative DRP1 protein level in panel b, one‐way ANOVA, *n* = 3. (g, h) Primary astrocytes isolated from *ALCAT1^−/−^* mice and *WT* controls (g) or SH‐SY5Y cells (h) were cultured in a medium supplemented with MPP^+^ (0.2 mM for astrocytes and 0.5 mM for SH‐SY5Y) or vehicle in the presence or absence of 10 μM of A320 for 24 hr, stained with MitoTracker Red, followed by confocal imaging analysis of mitochondrial architecture. Scale bar: 10 μm. (i, j) Quantification of mitochondrial architecture of panel g and h, respectively, *n* = 100 cells. Data are mean ± *SEM*; *p < 0.05, **p < 0.01, and ***p < 0.001

The onset of sporadic PD is associated with mitochondrial fragmentation, which is implicated in the pathogenesis of the disease (Santos, Esteves, Silva, Januario, & Cardoso, 2015). Mitochondrial architecture is regulated by a family of mitochondrial GTPases, including MFN2, OPA1, and DRP1, that regulate the mitochondrial fusion and fission process. MFN2 is depleted in PD patients, whereas DRP1 inhibition protects neurotoxicity (Rappold et al., 2014). Accordingly, we show that MPTP treatment not only downregulated MFN2 (Figure 5a, quantified in Supporting Information Figure S3e,f), but also promoted translocation of DRP1 from the cytoplasm to mitochondria, a key step required for mitochondrial fission (Figure 5b, quantified in Figure 5c–f), whereas MPTP treatment did not change S‐OPA1/L‐OPA1 ratio (Figure 5a, quantified in Supporting Information Figure S3c,d). In support of the role of ALCAT1 in the defects, ALCAT1 protein expression was significantly upregulated by MPTP treatment in the midbrain (Figure 5a, quantified in Supporting Information Figure S3a–c), whereas ALCAT1 deficiency or inhibition by A320 not only restored MFN2 and OPA1 protein expression (Figure 5a, quantified in Supporting Information Figure S3c,d), but also significantly attenuated the mitochondrial association of DRP1 in the midbrain of MPTP mice (Figure 5b, quantified in Figure 5c–f). Consequently, inhibition of ALCAT1 by A320 protected primary astrocytes and SH‐SY5Y cells from MPP^+^‐induced mitochondrial fragmentation (Figure 5g,h, quantified in Figure 5i,j).

### 2.7 ALCAT1 links MPP^+^‐induced oxidative stress to lipid peroxidation and mtDNA depletion

**Figure 6:**
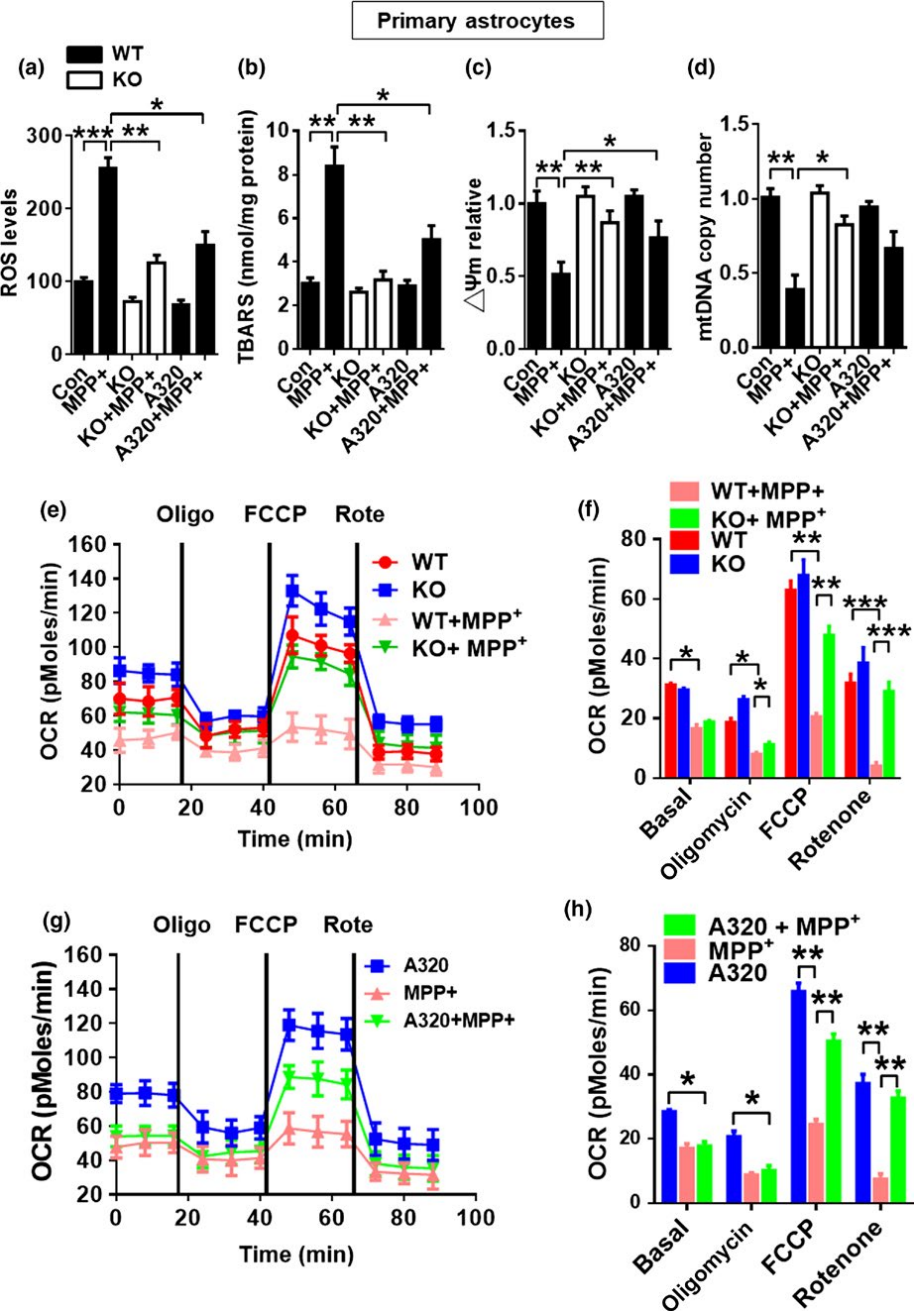
ALCAT1 deficiency or inhibition by A320 prevents oxidative stress, lipid peroxidation, mtDNA depletion, and defective respiration. Primary astrocytes isolated from *ALCAT1^−/−^* mice and *WT* controls were cultured in medium supplemented with MPP^+^ (0.2 mM) in the presence or absence of 10 μM of A320 for 24 hr, followed by analysis of mitochondrial dysfunction including (a) cellular reactive oxygen species production by using cell permeant reagent 2′,7′–dichlorofluorescin diacetate (DCFDA) kit and flow cytometry; (b) lipid peroxidation in the form of malondialdehyde (MDA) by using a thiobarbituric acid reactive substances (TBARS) assay kit; (c) mitochondrial membrane potential in isolated astrocytes stained with JC‐1 by confocal imaging analysis; (d) mtDNA copy numbers by real‐time PCR analysis; and (e, g) oxygen consumption rate (OCR) by Seahorse XF‐96 in response to treatment with indicated mitochondrial inhibitors, including oligomycin (Oligo), FCCP, and rotenone (Rote). (f–h) Quantification of OCR in panel e and g, respectively. Data are mean ± *SEM*; one‐way ANOVA, *n* = 5, *p < 0.05, **p < 0.01, and ***p < 0.001

Oxidative stress and mtDNA depletion are common defects in PD patients. We next questioned whether ALCAT1 deficiency protects mice from MPP^+^‐induced oxidative stress and mitochondrial dysfunction in primary astrocytes and in SH‐SY5Y cells. We chose primary astrocytes for the experiments, because they play an essential role in brain antioxidant defense mechanisms. MPP^+^ treatment caused severe oxidative stress, as evidenced by increased level of ROS in astrocytes stained with 2′‐7′‐dichlorodihydrofluorescein diacetate (DCFH‐DA), a chemiluminescent probe that directly measures the redox state of a cell (Figure 6a). ROS causes lipid peroxidation, which is also implicated in the pathogenesis of PD. Consistent with this notion, MPP^+^ treatment also increased production of thiobarbituric acid reactive substances (TBARS), a by‐product of lipid peroxidation (Figure 6b). In contrast, these defects were significantly attenuated by ALCAT1 deficiency or inhibition by A320. Consequently, ALCAT1 deficiency or inhibition by A320 also restored mitochondrial membrane potential (△Ψm) (Figure 6c) and mtDNA copy number which was depleted by MPP^+^ treatment in primary astrocytes (Figure 6d). Again, these defects were mitigated by ALCAT1 deficiency or inhibition by A320, suggesting a major role of ALCAT1 in regulating mitochondrial mass and mtDNA stability. These findings were further corroborated by similar results found in SH‐5YSY cells (Supporting Information Figure S4a–d).

### 2.8 ALCAT1 deficiency or pharmacological inhibition restores mitochondrial respiration

Mitochondria generate most of the cell's supply of ATP via the oxidative phosphorylation pathway, which can be measured by the oxygen consumption rate (OCR). Using a Seahorse XF‐96 extracellular flux analyzer, we next determined the effects of ALCAT1 and MPP^+^ treatment in regulating OCR in response to different inhibitors of the electron transport chain, including oligomycin (an ATPase inhibitor), FCCP (a mitochondrial uncoupler), and rotenone (a complex I inhibitor). The results showed that MPP^+^ treatment severely impaired mitochondrial respiration both in primary astrocytes (Figure 6e, quantified in Figure 6f) and in SH‐SY5Y cells (Supporting Information Figure S4e, quantified in Figure S4f). In contrast, ALCAT1 deficiency or treatment with A320 restored mitochondrial respiration in response to MPP^+^ treatment both in primary astrocytes (Figure 6e,g, quantified in Figure 6f,h) and in SH‐SY5Y cells (Supporting Information Figure S4e, quantified in Figure S4f). Together, these findings further support a causative role of ALCAT1 in mitochondrial dysfunction associated with MPTP‐induced neurotoxicity.

## 3 DISCUSSION

Evidence linking mitochondrial dysfunction to PD arose in the late 1970s when accidental exposure to MPTP was found to cause Parkinsonism and DA neurodegeneration (Langston, Ballard, Tetrud, & Irwin, 1983). Using mice with targeted deletion of ALCAT1 and a novel chemical inhibitor of the enzyme, we investigated a role of pathological remodeling by ALCAT1 in the MPTP‐induced neurotoxicity and degeneration of DA neurons. ALCAT1 is the first acyltransferase that catalyzes the pathogenic remodeling of CL with very long polyunsaturated fatty acids, leading to CL peroxidation and depletion in several aging‐related diseases (Li et al., 2010; Liu et al., 2012; Wang et al., 2015). We showed in this study that the onset of MPTP‐induced PD caused severe oxidative stress, lipid peroxidation, and mitochondrial dysfunction. These defects were mitigated by targeted deletion of ALCAT1, which also prevented MPTP‐induced behavioral impairments, neurotoxicity, and depletion of DA neurons. These findings were further corroborated by treatment of mice with an ALCAT1 inhibitor which restored mitochondrial function and prevented MPTP‐induced neurotoxicity and cell death. In further support of the findings, ALCAT1 deficiency or inhibition also prevented apoptosis in the midbrain by downregulating the expression of cleaved caspase‐3, Bax, and NLRP3 concurrently with upregulated Bcl2 expression in the midbrain.

Mitophagy plays an essential role in eliminating damaged mitochondrial from oxidative stress. Accordingly, defective mitophagy is part of the pathogenesis of PD, as evidenced by rare forms of PD in human patients caused by genetic mutations of PINK1 and Parkin, key regulators of mitophagy (Chen & Dorn, 2013). CL is also required for multiple processes of mitophagy in addition to its pivotal role in supporting mitochondrial oxidative phosphorylation (Hsu et al., 2015; Hsu & Shi, 2017). Among all phospholipids, CL is extremely sensitive to oxidative damage by ROS due to its high content in polyunsaturated fatty acids and exclusive location inside mitochondria where ROS are generated. CL oxidation by ROS stimulates its externalization to the mitochondrial surface, which not only triggers CL remodeling at the mitochondria‐associated membrane (MAM) by ALCAT1, but also serves as the key recognition signal for mitophagy (Chu et al., 2014; Hsu & Shi, 2017). Consequently, we showed in this study that ablation or pharmacological inhibition of ALCAT1 significantly improved mitophagy by promoting Parkin association with mitochondria, lipidation of LC3, and autophagic consumption of p62, leading to restoration of mitochondrial respiration in MPTP mice.

The onset of MPTP‐induced PD is associated with mitochondrial fragmentation, a common defect associated with other aging‐related diseases, but the underlying causes remain elusive. CL is required for the activity of multiple enzymes involved in the mitochondrial fission and fusion process which plays a key role in mitochondrial quality control process (Chu et al., 2014; Hsu & Shi, 2017). Mitochondrial fusion allows functional complementation of partially dysfunctional mitochondria, whereas the fission process is required for the isolation of damaged mitochondria to be eliminated through mitophagy (Twig et al., 2008). MFN2 is a mitochondrial GTPase required for mitochondrial fusion by tethering two mitochondria as a functional bridge (de Brito & Scorrano, 2008). Therefore, MFN2 deficiency dramatically impairs starvation‐induced autophagy (Hailey et al., 2010). MEFs from *MFN2^−/−^* mice also display fragmented mitochondria and dilated MAM that resemble the defects caused by ALCAT1 overexpression (Li et al., 2012). Using isolated brain tissue from *ALCAT1^−/−^*and the *WT* control mice, we showed that MPTP treatment caused mitochondrial fragmentation by inhibiting MFN2 expression and by promoting the association of DRP1 with mitochondria, a key step required for mitochondrial fission. The defects were mitigated by ALCAT1 depletion or pharmacological inhibition, lending further support to our hypothesis that upregulated ALCAT1 expression by MPTP links mitochondrial fragmentation and defective mitophagy to mitochondrial dysfunction.

Dysfunction of mitochondrial complex I contributes to the pathogenesis of human PD patients as well as MPTP‐induced neurotoxicity in mice (Przedborski, 2017; Schapira, Cooper et al., 1990; Schapira, Mann et al., 1990). Although the precise causes for the defect are yet to be fully elucidated, oxidative damage of CL is also implicated in the pathogenesis. Accordingly, inhibition of complex I activity by MPTP is accompanied by an increased production of CL peroxides (Perier et al., 2005). Deficits of complex I also stimulate intramitochondrial oxidative stress, further exacerbating oxidative damage of CL. As a consequence of the vicious cycle, oxidized CL dissociates from cytochrome c, triggering the onset of apoptosis (Lutter et al., 2000; Petrosillo, Ruggiero, & Paradies, 2003). In further support of a causative role of upregulated ALCAT1 expression in MPTP‐induced neurotoxicity, MPTP treatment significantly increased ROS production and lipid peroxidation, which is significantly attenuated by ALCAT1 depletion and by pharmacological inhibition. The findings are further corroborated by our previous report that ALCAT1 overexpression significantly decreased mitochondrial complex I activity, whereas ALCAT1 deficiency significantly increased complex I activity (Li et al., 2010).

ROS and lipid peroxides promote oligomerization of α‐synuclein through covalent modifications, which is implicated in the pathogenesis of PD and other neurological diseases (Souza et al., 2000). Accordingly, primary fibroblasts isolated from PD patients exhibit increased lipid peroxidation, enhanced sensitivity to oxidative stress, and impaired respiratory activity (Hoepken et al., 2007). A recent study implicated a key role of CL in regulating autophagic degradation of aggregated α‐synuclein with PD (Ghio et al., 2016). Consistent with this notion, we showed MPTP not only upregulated α‐synuclein expression, but also promoted α‐synuclein oligomerization and S‐129 phosphorylation, a hallmark of PD. In final support of a key role of ALCAT1 in MPTP‐induced neurotoxicity, we showed that ALCAT1 deficiency or pharmacological inhibition prevented α‐synuclein oligomerization and S‐129 phosphorylation, whereas transgenic expression of human α‐synuclein dramatically upregulated ALCAT1 expression in the brain, implicating a vicious cycle. Consistent with the findings, both ALCAT1 and α‐synuclein are located at the MAM, the primary site for autophagosome biogenesis and phospholipid remodeling (Hsu & Shi, 2017). Overexpression of ALCAT1 disrupts the MAM (Li et al., 2012), which is reminiscent of the defect caused by mutation of α‐synuclein (Guardia‐Laguarta et al., 2014). Together, these findings identified a key role of the ALCAT1 enzyme in MPTP‐induced neurotoxicity, suggesting that targeting ALCAT1 with molecule inhibitors may provide a novel treatment for PD, a debilitating disease without any effective treatment.

## 4 EXPERIMENTAL PROCEDURES

### 4.1 Animal care

The study protocol was approved by the Institutional Animal Care and Use Committee of Nanjing Medical University. Mice with targeted deletion of the ALCAT1 gene were generated as previously described (Li et al., 2010). The mouse model of PD (10 weeks) was generated by intraperitoneally injection with MPTP hydrochloride (30 mg/kg) (Sigma‐Aldrich, St. Louis, MO, USA, #M0896) for 7 days and A320 was given i.p. 1 mg/kg 3 days prior to the first dose of MPTP for a total of 13 days, followed by behavioral tests on day 14. After the behavioral tests, the mice were sacrificed, and the brain tissues were collected for further analysis.

### 4.2 Antibodies and reagents

Antibodies used in the present studies included polyclonal antibodies to optic atrophy 1(OPA1) (80471S), LC3 (12741S), p62 (5114S), DRP1 (8570S), voltage‐dependent anion channel (VDAC) (4866S), cleaved caspase‐3 (9661S), Bax (2772S), Bcl‐2 (2876S), and PINK1 (6946S), all of which were purchased from Cell Signaling (Danvers, MA, USA). Mitofusin‐2 (MFN2) (Ab56889), Parkin (Ab15954), α‐synuclein (Ab1903), and phosphor S129 α‐synuclein (Ab51253) were purchased from Abcam (Cambridge, UK). TH (T1299), NeuN (ABN90), and β‐actin (A5441) antibodies were purchased from Sigma‐Aldrich. CCK8(C0038) was purchased from Beyotime Biotechnology (Shanghai, China). GFAP (SC‐71143) and Tom 20 (SC‐136211) antibodies were purchased from Santa Cruz Biotechnology (Santa Cruz, CA, USA). A320 (ALCAT1 inhibitor) was obtained from Perenna Pharmaceuticals Inc (San Antonio, TX, USA). ALCAT1 antibody was obtained from Dr. Arai (University of Tokyo, Japan).

### 4.3 Cell culture and drug treatments

SH‐SY5Y cells (ATCC) were maintained in DMEM medium containing 10% FBS and antibiotics. The cells cultures were pre‐exposed to different concentrations of A320 for 30 min, then treated with MPP^+^ (0.5 mM) (Sigma‐Aldrich, #D048) for 24 hr, and then processed for further analysis. Primary cortical astrocytes were isolated, cultured in DMEM/F12 supplemented with 10% FBS, and were pre‐exposed to different concentrations of A320 for 30 min prior to the addition of MPP^+^ at a final concentration of 0.2 mM, and analyzed for oxidative stress levels, mitochondrial respiration, and mitochondrial dynamics. Immunofluorescence staining and confocal imaging analysis were carried out as previously described (Hsu et al., 2015)**.**

### 4.4 Behavior tests

In these double‐blind experiments, neither the participants nor the researchers knew which participants belonged to the vehicle group, nor the test group. *Open field test: *Horizontal locomotor activity was measured using an infrared photobeam activity cage system (ANY‐maze video tracking software, Stoelting, USA). Before the experiment, the animals were habituated in the test room for 30 min. Then, each mouse was placed in a clear, open‐top, square Plexiglas box (30 × 30 × 40 cm^3^) in a secluded room and allowed to freely explore for 6 min. Rearing number and traveled distance were measured within 6 min. *Rotarod test: *One day before testing, the mice were trained until they could remain on the rotarod for 120 s without falling. During testing, mice were placed on the rotarod, and the rotation speed was increased from 5 to 40 r/min over 5 min. The latency (time) to fall from the rotarod was automatically recorded. Each mouse was tested in three independent trials with a 20‐min intertrial interval. The latency to fall was calculated as the average of three trials. *Pole test:* Mice were placed head down on the top of a vertical wooden pole (60 cm in length and 2 cm in diameter) with a rough surface. The latency for the mice to climb down from the top of the pole to the base was then measured. Trials were considered a failure if the mouse jumped or slid down the pole. Each mouse underwent five trials, and the average latency was then recorded. *Beam walking test:* Challenging beam was a 1‐m‐long wooden beam suspended 23 cm above a bench top, which was covered with soft pads to protect the mouse in case of a fall. The beam was divided into four gradually narrowing sections (25 cm/section) leading to the mouse's home cage. All mice were pretrained for two consecutive days on traversing the beam. On the third day, each mouse was given five trials (intertrial intervals = 10–12 s), and the average time was calculated.

### 4.5 Immunofluorescence staining and cell counting

Following the behavioral tests, mice were anesthetized with 4% chloral hydrate and then perfused through the left ventricle with phosphate‐buffered saline (PBS, pH 7.4), followed by paraformaldehyde (4%) in 0.1 M PBS (pH 7.4). Following perfusion, the mice were sacrificed, and their brains were collected and postfixed in paraformaldehyde overnight at 4°C, followed by 30% sucrose overnight at 4°C. The brains were serially cut into 20‐µm coronal sections using a freezing microtome. For immunofluorescence staining, the midbrain sections were rinsed three times with 0.1 M PBS and incubated in PBS containing 3% BSA and 0.3% Triton for 2 hr at room temperature. The sections were washed in PBS and then incubated with anti‐TH antibody or anti‐NeuN antibody at 4°C for 24 hr. The sections were washed (3 × 10 min) in PBST (PBS containing 0.3% Triton) and incubated with Alexa Fluor 488 conjugated goat anti‐rabbit IgG for 2 hr at room temperature in the dark. The sections were washed (10 min each) and then mounted using mounting medium and imaged using a laser confocal fluorescent microscope. For cell quantification in vivo studies, the numbers of TH‐, NeuN‐immunoreactive cells in the SNcp of the midbrain were assessed using the optical fractionator (Stereo Investigator 7; MBF Bioscience, Williston, VT, USA). The sampling scheme was designed to have a coefficient of error <10% in order to get reliable results. The total numbers of immunoreactive cells in the entire SNcp were counted from five mouse brains per group. Each brain was sampled with six serial sections at six intervals for the staining. Counting was performed blinded to the treatment history.

### 4.6 Mitochondrial assays

Cells were treated with MPP^+^ (0.5 mM for SH‐SY5Y cells and 0.2 mM for astrocytes) and A320 (A320, 10 μM, was added 30 min before MPP^+^) for 24 hr. Cells were then stained with MitoTracker Red (final concentration, 100 nM) for 12 min in a 37°C incubator and washed with PBS three times, 5 min each wash. The SH‐SY5Y cells were then analyzed under confocal microscopy. mtDNA copy number was analyzed by methods as previously described (Wang et al., 2015). Mitochondrial membrane potential (△Ψm) was assessed with the fluorescent probe JC‐1 (Thermo Fisher Scientific, Waltham, MA, USA, #T3168). Cells were incubated with JC‐1 (5 µM) for 10 min at 37℃, washed, and placed on a thermostated stage at 37℃. Fluorescent images were visualized by a flow cytometer with excitation at 490 nm and emission at 520 nm. The ratio of J‐aggregate to JC‐1 monomer intensity for each region was calculated. A decrease in this ratio was interpreted as loss of △Ψm, whereas an increase in the ratio was interpreted as gain in △Ψm.

### 4.7 mtDNA copy number Assays

For mtDNA isolation, midbrain tissue was homogenized and a mitochondrial fraction was isolated as previously described (Wang et al., 2015). Analysis of mitochondrial copy number in kidney tissue was carried out using mitochondrion‐encoded NADH dehydrogenase 1 (ND1) as the mtDNA marker and cyclophilin A as a genomic marker. Quantitative PCR amplification was carried out using the following programs: step 1, 95°C for 10 min; step 2, 95°C for 15 s; step 3, 60°C for 1 min; step 4, repeat step 2 and step 3 forty times; and step 5, melt curve from 65°C to 95°C. The primer pairs used included the following: ND1 (forward, 5′‐ TGACCCATAGCCATAATATGATTT‐3′; reverse, 5′‐CTCTACGTTAAACCCTGATACTAA‐3′) and cyclophilin A (forward, 5′‐ACACGCCATAATGGCACTCC‐3′; reverse, 5′‐ CAGTCTTGGCAGTGCAGAT‐3′).

### 4.8 Statistical analysis

Data were analyzed using GraphPad Prism software (version 6.0) and expressed as a mean ± *SEM*. Two‐way and one‐way ANOVAs followed by Bonferroni post hoc tests were utilized for multiple‐group comparisons. Student's *t*‐test was used for comparisons between two groups, and statistical significance was considered at *p* < 0.05.

## CONFLICT OF INTEREST

Y.S. is a shareholder of Perenna Pharmaceuticals Inc, a privately held company which provided the ALCAT1 inhibitor A320 used in this study.
