# Supplementary figures and images for: Cardiolipin remodeling by ALCAT1 links mitochondrial dysfunction to Parkinson’s diseases
Source: Aging Cell. 2019 Mar 5;18(3):e12941. doi: 10.1111/acel.12941 (PMC6516155; doi:10.1111/acel.12941)

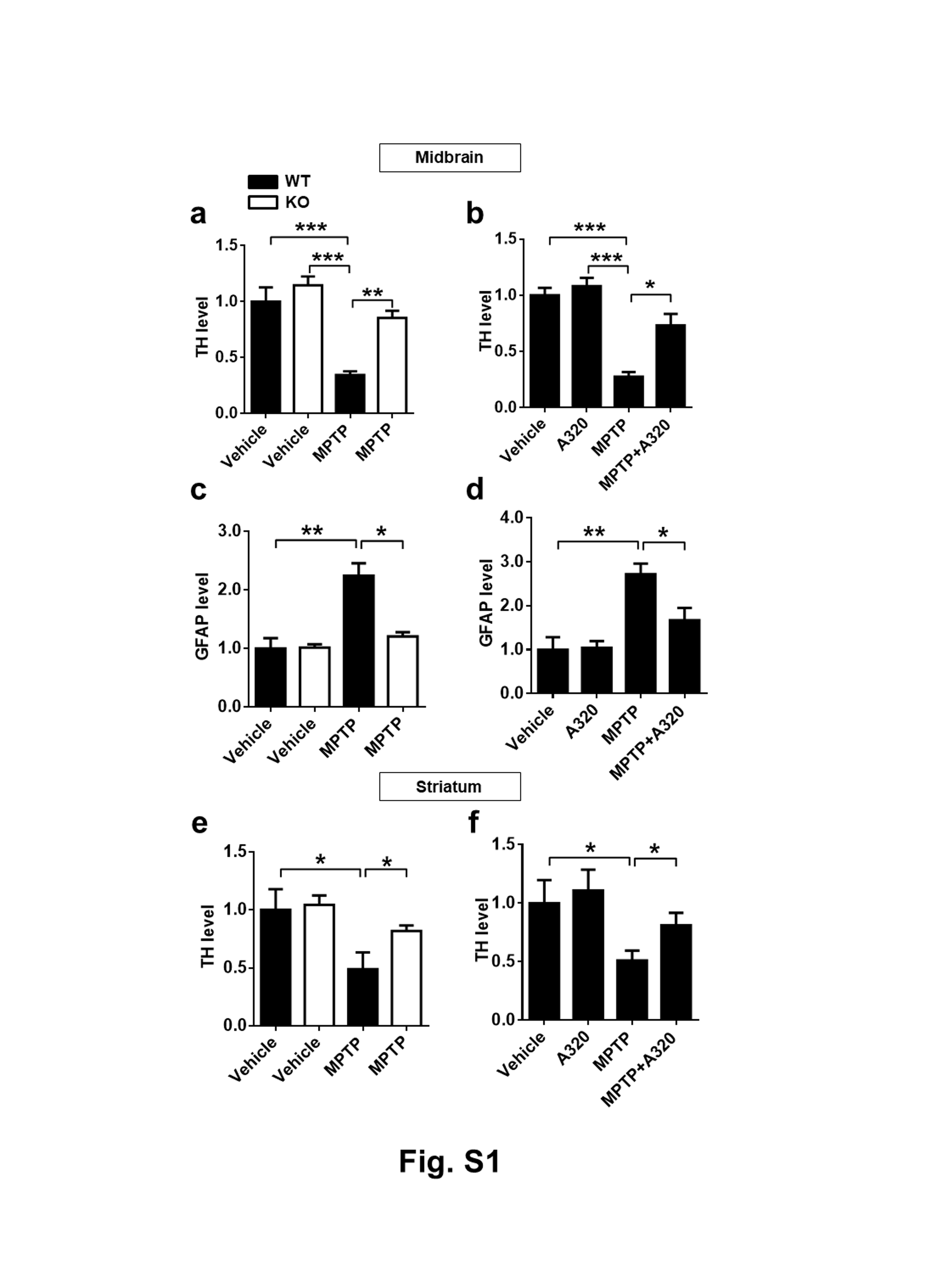

Supplement: Supplementary file 1 [file ACEL-18-e12941-s001.tif]

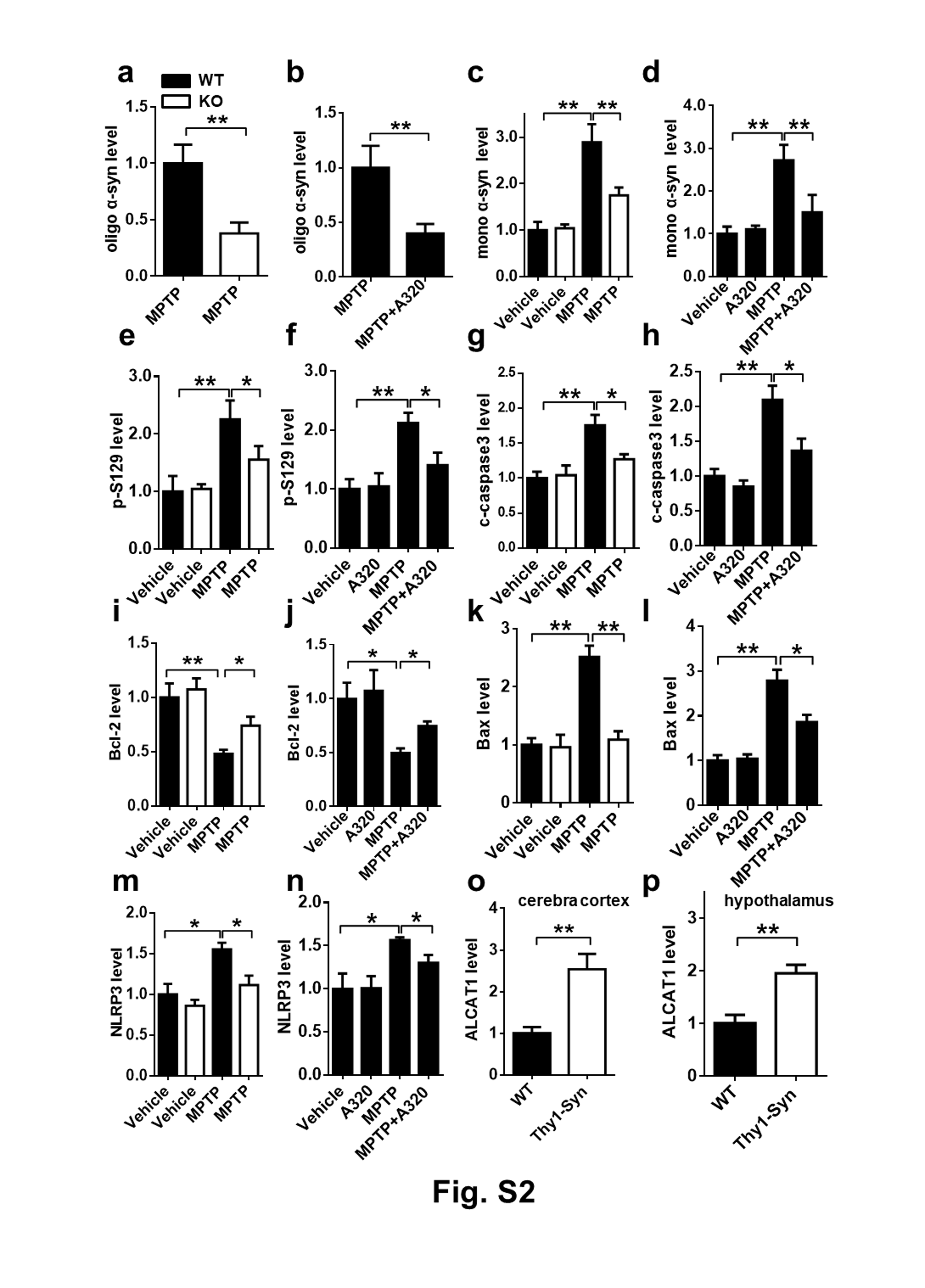

Supplement: Supplementary file 2 [file ACEL-18-e12941-s002.tif]

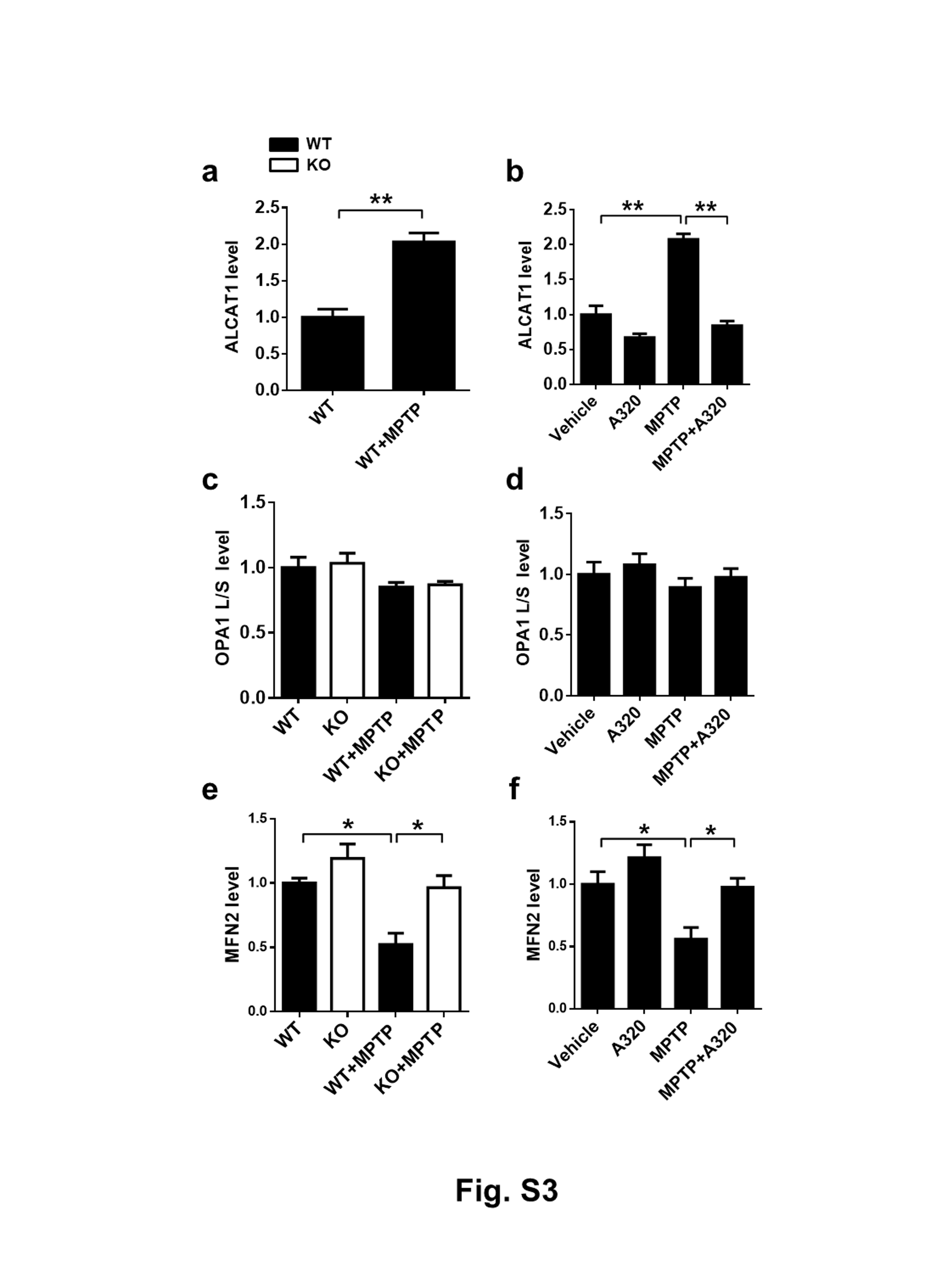

Supplement: Supplementary file 3 [file ACEL-18-e12941-s003.tif]

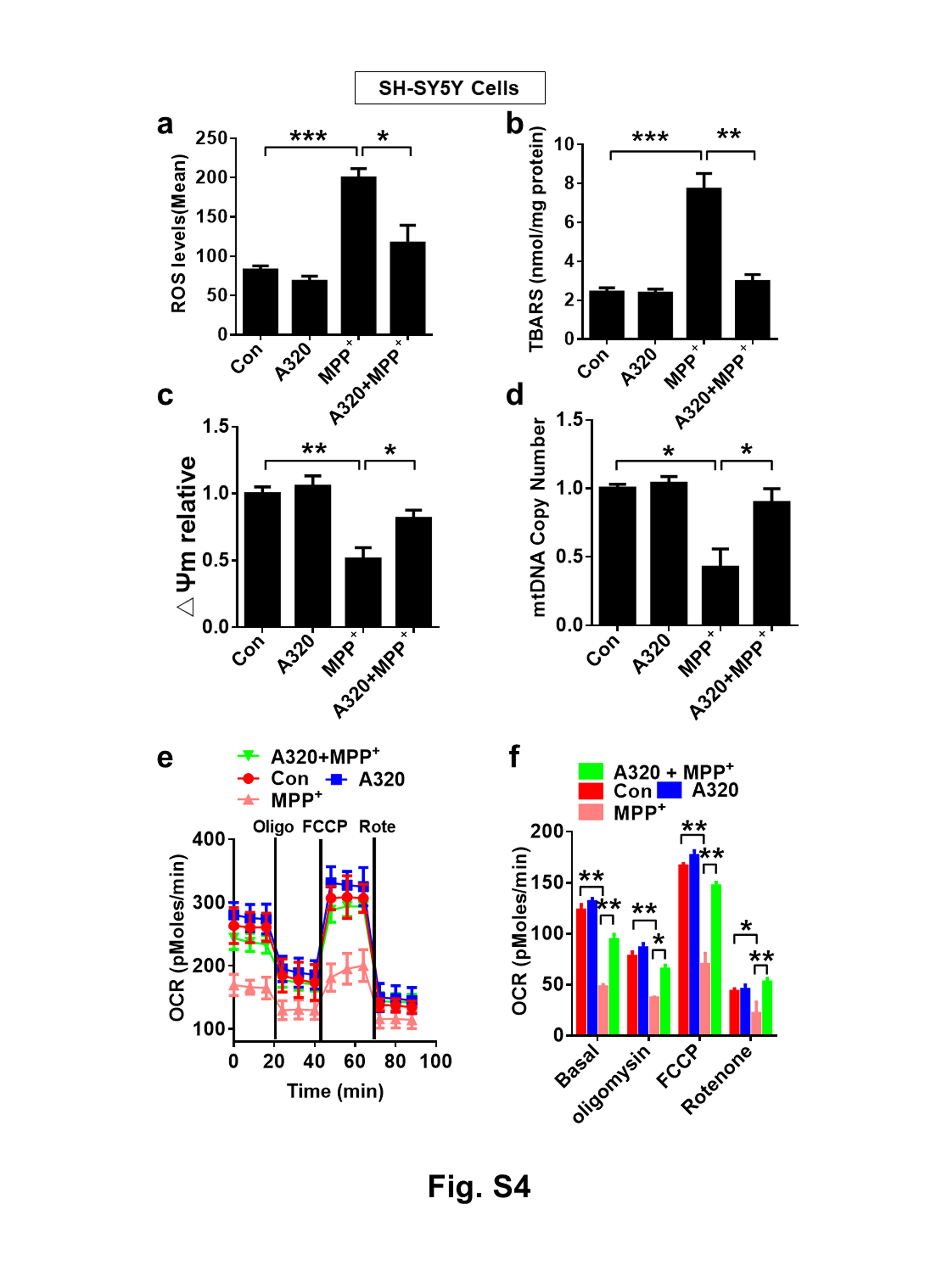

Supplement: Supplementary file 4 [file ACEL-18-e12941-s004.tif]
