# Supplementary material for: Cardiolipin remodeling by ALCAT1 links mitochondrial dysfunction to Parkinson’s diseases
Source: Aging Cell. 2019 Mar 5;18(3):e12941. doi: 10.1111/acel.12941 (PMC6516155; doi:10.1111/acel.12941)
Supplement: Supplementary file 5 [file ACEL-18-e12941-s005.docx]

**Supplementary Methods**

**Measurement of mitochondrial bioenergetic function by extracellular flux (XF) analysis.** The XF96 Extracellular Flux Analyzer (Seahorse Bioscience, Billerica, MA, USA) was used to measure the rate changes in extracellular flux of dissolved O_2_ and protons in the medium immediately surrounding the adherent intact cells cultured on a XF96-well microplate (Seahorse Bioscience). The SH-SY5Y cells or primary astrocytes were seeded in XF96-well microplates at 1.0×10^4^ cells/well (0.32 cm^2^) in 100 µL DMEM or DMEM/F12 medium with 10% FBS and incubated at 37°C with 5% CO_2_ for 24 h. The SH-SY5Y cells or primary astrocytes were then treated as described above. The medium was removed and replaced with assay medium 1 h prior to the beginning of the assay and maintained at 37°C. the mitochondrial oxygen consumption rates (OCRs) were measured using the XF Cell Mito Stress Test kit (Seahorse Bioscience), and the following were sequentially added to each well: oligomycin (1 µM, blocker of the mitochondrial complex V, inhibiting the electron chain from being coupled to ATP synthesis), FCCP (3 µM, an uncoupling agent which allows maximum electron transport) and rotenone (1 µM, mitochondrial complex I blocker which eliminates mitochondrial respiration).

**CCK8 assay.** Cells were seeded in 96-well plates at a density of 4000 cells per well. After 12 h, MPP^+^ and different concentrations of A320 (A320 was added 30 min before MPP^+^) were added to the wells (three replicates for each group) for 24 h. Briefly, the cells were rinsed with PBS, and 10 μL CCK8 was added to each well. The plates were incubated at 37°C for an additional 1 h. The absorbance was measured at 450 nm using a spectrophotometer.

**Measurement of ROS and MDA.** Formation of ROS was evaluated using 2’, 7’-dichloroﬂuorescein di-acetate (DCFH-DA, Sigma), a membrane-permeable probe de-esteriﬁed intracellularly. Upon oxidation by ROS, DCFH yields the highly ﬂuorescent product dichloroﬂuorescein. After treatment of listed reagents for 3 h, cells were loaded with DCFH-DA (50 µM) in Dulbecco’s modiﬁed Eagle’s medium for 60 min in the dark and ﬁxed with 4% formaldehyde. After rinsing cells twice with PBS, ﬂuorescence was read at the excitation wavelength of 488 nm and the emission wavelength of 530 ± 20 nm. DCFH-DA was initially dissolved to 40–50 mM in ethanol and stored at -20℃. A ﬁnal concentration of ethanol (0.6% or less) did not alter the ﬂuorescence measurement. Lipid peroxidation products in the midbrain tissue were quantified by measuring the level of Thiobarbituric Acid Reactive Substances (TBARS) in the form of malondialdehyde (MDA) using a TBARS kit (Cayman Ann Arbor, MI, USA, #10009055) according to manufacturer’s instruction.

**Isolation of mitochondrial-enriched fraction and lysate preparation.** Brain tissue from SN or SH-SY5Y cells were harvested and homogenized in the mitochondrial isolation buffer (250 mM sucrose, 20 mM HEPES-NaOH, pH 7.5, 10 mM KCl, 1.5 mM MgCl_2_, 1 mM EDTA, protease inhibitor cocktail, phosphatase inhibitor cocktail). The homogenates were spun at 800 g for 10 min at 4 °C and the resulting supernatants were spun at 10,000 g for 20 min at 4 °C. The pellets were then washed with lysis buffer and spun at 10,000 g again for 20 min at 4 °C. The final pellets were suspended in lysis buffer containing 1% Triton X-100 and were the mitochondrial-rich lysate fractions. The supernatants were spun at 100,000 g for 1 h and the final supernatants were thus cytosolic fractions. The mitochondrial membrane protein Tom20 was used as a marker and loading control. β-actin was used as a marker and a loading control for the cytosolic fractions.

**Western blot analysis.** Cells were washed twice with PBS after treatment and solubilized in RIPA lysis buffer. Tissue samples were homogenized in tissue lysis buffer. Protein concentrations were determined by the BCA method. Protein samples (10 µg) were separated by 10% SDS ⁄ PAGE and PVDF membrane. After blocking in 10% milk with TBS-T buffer (10 mM Tris–HCl, 120 mM NaCl, 0.1% Tween-20, pH 7.4) for 1 h at room temperature, the membrane was incubated with various primary antibodies (1:1000) at 4 °C overnight. Membranes were then washed three times in TBST buffer, followed by incubation with 1:10000 dilutions of HRP conjugated anti-rabbit/mouse IgG at room temperature for 1 h, and washed three times in TBST. Visualization was carried out using an ECL (advanced chemiluminescence) kit (GE Healthcare, Bucks, UK). The density of the bands on the Western blots was quantified by densitometric analysis of the scanned blots using ImageJ software. Different primary antibodies OPA1 (1:1000), LC3 (1:1000), p62 (1:1000), DRP1 (1:1000), VDAC (1:1000), cleaved caspase-3 (1:1000), Bax (1:1000), Bcl-2 (1:1000), PINK1 (1:1000), MFN2 (1:1000), Parkin (1:1000), α-synuclein (1:1000), phosphor S129 α-synuclein (1:1000), TH (1:4000), β-actin (1:2000), GFAP (1:1000), Tom 20 (1:1000), ALCAT1 (1:1000) were used in the present study .

**SI Figure legends**

**FIGURE S1.** Quantitative analysis of data presented in Figure 2. (a-d) Quantitative analysis of relative protein expression levels of TH and GFAP in midbrain. (e-f) Quantitative analysis of relative TH levels in striatum. Data are Mean (% of controls) ± SEM, one-way ANOVA, n = 3, **P*<0.05, ***P*<0.01, and ****P*<0.001

**FIGURE S2.** Quantitative analysis of data presented in Figure 3. (a-n) Quantitative analysis of relative protein levels of α-synuclein oligomers (a-b), α-synuclein monomers (c-d), α-synuclein phosphorylation at S129 (e-f), cleaved caspase-3(g-h), Bcl-2 (i-j), Bax (k-l), and NLRP3 (m-n) in the midbrain. (o-p) quantitative analysis of relative ALCAT1 protein expression in cerebral cortex (o) and hypothalamus (p). Data are Mean ± SEM, one-way ANOVA, n = 3, **P*<0.05, ***P*<0.01, and ****P*<0.001.

**FIGURE S3.** Quantitative analysis of data presented in Figure 5, including ALCAT1 (a-b), OPA1 (c-d), and MFN2 (e-f) in the midbrain. Data are Mean ± SEM, one-way ANOVA, n = 3, **P*<0.05, ***P*<0.01, and ****P*<0.001.

**FIGURE S4.** Inhibition of ALCAT1 by A320 prevents oxidative stress, mtDNA depletion, and defective respiration. SH-SY5Y cells were cultured in medium supplemented with MPP^+^ (0.5 mM) in the presence or absence of A320 (10 μM) for 24 h, followed by analysis of mitochondrial dysfunction including: (a) Cellular ROS were measured by using cell permeant reagent 2’,7’–dichlorofluorescin diacetate (DCFDA) kit and ﬂow cytometry. (b) Lipid peroxidation products were measured in the form of malondialdehyde (MDA) by using a TBARS assay kit. (c) Mitochondrial membrane potential was measured in isolated astrocytes stained with JC-1 by confocal imaging analysis. (d) mtDNA copy numbers were analyzed by real time-PCR analysis. (e) Mitochondrial respiration was analyzed by Seahorse XF-96 by measuring changes in oxygen consumption rate (OCR) in response to treatment with indicated mitochondrial inhibitors, including oligomycin (Oligo), FCCP, rotenone (Rote). (f) Quantification of OCR in panel e. Data are Mean ± SEM; one-way ANOVA, n=5, **P*<0.05, ***P*<0.01, and ****P*<0.001.
